# Syndromic surveillance performance in Canada throughout the COVID-19 pandemic, March 1, 2020 to March 4, 2023

**DOI:** 10.14745/ccdr.v49i1112a06

**Published:** 2023-11-01

**Authors:** Myriam Ben Moussa, Abbas Rahal, Liza Lee, Shamir Mukhi

**Affiliations:** 1 Centre for Emerging and Respiratory Infections and Pandemic Preparedness, Public Health Agency of Canada, Ottawa, ON; 2 Canadian Network for Public Health Intelligence, National Microbiology Laboratory, Edmonton, AB

**Keywords:** syndromic surveillance, participatory surveillance, influenza, respiratory viruses

## Abstract

The coronavirus disease 2019 (COVID-19) pandemic has highlighted the need for robust surveillance of respiratory viruses. Syndromic surveillance continues to be an important surveillance component recommended by the World Health Organization (WHO). While FluWatchers, Canada's syndromic surveillance system, has been in place since 2015, the COVID-19 pandemic provided a valuable opportunity to expand the program's scope and underlying technology infrastructure. Following some structural changes to FluWatchers syndromic questionnaire, participants are now able to contribute valuable data to the non-specific surveillance of respiratory virus activity across Canada. This article examines the performance of FluWatchers' syndromic surveillance over the three years of the COVID-19 pandemic in Canada. More specifically, this article examines FluWatchers' performance with respect to the correlation between the FluWatchers influenza-like illness (ILI) and acute respiratory infection (ARI) indicators and total respiratory virus detections (RVDs) in Canada, including influenza, respiratory syncytial virus (RSV), severe acute respiratory syndrome coronavirus 2 (SARS-CoV-2), and other respiratory viruses.

## Introduction

FluWatchers is a participatory syndromic surveillance system that has been in place in Canada since 2015. This crowdsourced system, which relies on volunteers in Canada to report influenza-like illness (ILI) symptoms on a weekly basis, was initially implemented to monitor influenza as part of the FluWatch program ((1)). Respiratory viruses, including both influenza and non-influenza viruses such as respiratory syncytial virus (RSV), severe acute respiratory syndrome coronavirus 2 (SARS-CoV-2), and others place a substantial burden on global healthcare systems ((2,3)). In Canada alone, influenza is estimated to cause 12,200 hospitalizations and 3,500 deaths each year, based on pre-pandemic data ((3)). Readily available and robust surveillance data facilitates response and assists public health authorities in coordinating the implementation of public health measures, such as seasonal vaccination campaigns, to reduce the stress on the healthcare system. The collection of syndromic data aims not only to facilitate the early detection of epidemics and allow for the detection of unexpected circulation patterns, but also to pivot toward non-specific disease surveillance. Participatory syndromic surveillance has been internationally accepted as a robust supplement to traditional respiratory virus surveillance systems for over a decade. Programs similar to FluWatchers are in place across the globe, including FluTracking in Australia, and CoughWatchSA in South Africa ((4,5)). The InfluenzaNet network in Europe, in place since 2003, gathers and reports data from several European countries ((6)). A 10-year review of the system found that syndromic surveillance data for these countries correlated well with ILI incidence reported by the European Centre for Disease Prevention and Control ((7)). Notably, international syndromic surveillance systems also seem to be shifting toward non-specific respiratory infection surveillance. For instance, Outbreaks Near Me, formerly known as Flu Near You and COVID Near You, is a system in place in the United States that pivoted toward non-specific respiratory infection surveillance in December 2020 ((8)).

Valid surveillance-based indicators are essential to informing emerging infectious disease readiness and responses, by allowing the detection of signals of unusual or unexpected activity and the observation of epidemic dynamics in real time, notably in a post-pandemic context. Syndromic surveillance systems in the context of ILI and related illnesses must be adaptable and exhibit the potential for scalability in response to global epidemiological events. FluWatchers had long been known to be successful in its original intent of tracking influenza activity in Canada ((9)). However, throughout the COVID-19 pandemic, crowdsourced data had been assessed as a potential mitigation tool ((10)). As such, the program pivoted toward the inclusion of COVID-19 related data. With the increasing push toward integrative surveillance (11), FluWatchers could demonstrate potential to expand toward the non-specific syndromic surveillance of respiratory viruses in Canada. This approach might offer valuable insights into the prevalence and trends of such viruses while allowing for a broader perspective on health monitoring.

This descriptive surveillance study aimed to examine trends in syndromic indicators of ILI as compared to laboratory-confirmed respiratory virus detections (RVDs), including SARS-CoV-2, influenza, RSV, and all other respiratory viruses (ORVs) throughout the three years of the COVID-19 pandemic. This study also aimed to provide insight on FluWatchers population characteristics, in order to facilitate inferences on community circulation trends and assess how the population's evolution may contribute to syndromic surveillance performance.

## Methods

FluWatchers consists of an open cohort of volunteer participants located in Canada, who can both enroll and unsubscribe from participating at any time. Individuals within the Canadian population can enroll to participate online ((12)). Participants are eligible if they provide a valid Canadian postal code (first three digits) and a valid email address. At the time of enrolment, participants are also invited to enroll and report on behalf of their household members (13).

### FluWatchers data collection

FluWatchers data consists of self-reported weekly episodes of cough and/or fever. At enrolment, participants self-report their year of birth, their gender, the first three digits of their postal code of residence, their email address, and, if they choose, household members ((13)). Each week, participants receive an emailed invitation to participate in an online survey that asks whether they have experienced a cough or a fever in the previous week. Participants are also asked to provide input on their annual influenza vaccination status. Each weekly questionnaire must be responded to in full by each participant choosing to respond in a given week, and incomplete questionnaires are automatically excluded from that week's data. The number of questions a participant must respond to is dependent on whether the participant has reported a cough or a fever in a given week.

With the progression of the COVID-19 pandemic and the acquisition of additional evidence on its modes of transmission and symptomology, additional COVID-19-related questions were added to the follow-up questions ((1)), namely to capture the following COVID-19-related symptoms: shortness of breath (week of March 29, 2020), headache (week of April 19, 2020), skin rash, and runny/stuffy nose, loss of taste or smell (week of October 4, 2020). As of February 28, 2021, the following COVID-19 vaccination question was included: “Between December 2020 and now: Did you receive vaccination for COVID-19? [Yes/No].” Prior to April 2022, additional questions about other symptoms of illness experienced, absenteeism from work or school, and healthcare utilization were only asked from participants who reported cough and fever. Due to the variety of symptoms reported early in the COVID-19 pandemic, the questionnaire was modified to collect additional data from participants reporting only cough and/or fever. These substantial changes were swiftly executed in collaboration with the Canadian Network for Public Health Intelligence (CNPHI) team, the platform behind FluWatchers. Leveraging the technology's inherent agility and foresight, the evolution involved meticulous adjustments to the questionnaire and data handling processes. This ensured seamless integration for surveillance objectives while upholding user-friendliness.

In the spring of 2020, in response to the COVID-19 pandemic, the FluWatchers program initiated a campaign to increase participant enrolment. Additional participants were recruited via social media beginning on April 3, 2020. This recruitment consisted of five posts to the Healthy Canadians Facebook page, 23 posts to the Health Canada and Public Health Agency of Canada (PHAC) Twitter account (@GovCanHealth), and 14 posts to Canada's Chief Public Health Officer's official Twitter account (@CPHO_Canada). Furthermore, through an email campaign, existing participants were encouraged to invite others to participate in the FluWatchers program. These recruitment efforts, as well as the ability of participants to share links, resulted in an increase of 330% over a one year period, from 4,895 to 21,040 registered participants.

### Laboratory-based respiratory virus data

Data from FluWatchers was compared against laboratory-confirmed SARS-CoV-2, influenza and other seasonal respiratory viruses (adenovirus, coronavirus, enterovirus/rhinovirus, human metapneumovirus [hMPV], parainfluenza and RSV). Data on the number of tests positive for SARS-CoV-2 at a national level are collected by the Public Health Agency of Canada through reports by health authorities in the provinces and territories ((14)). The SARS-CoV-2 data used for this analysis were extracted from the PHAC COVID-19 Health InfoBase and contained information on cases with onset up to March 4, 2023, based on data reported up to June 21, 2023. Data on the number of tests positive for influenza and ORV are collected through PHAC's Respiratory Virus Detection Surveillance System (RVDSS) ((15)), which RVDSS collects weekly nucleic acid testing results from provincial, regional, and some hospital labs across Canada. Case data are subject to possible reporting delays between time of case notification to the provincial/territorial public health authority and time of reporting to PHAC.

### Measures

For each week, the weekly percentage of FluWatchers participants reporting either A) cough and fever, or B) cough or fever among all participants was calculated. For the purposes of this analysis, reports of cough and fever are referred to as ILI, and reports of cough or fever are referred to as acute respiratory infection (ARI), as they are intended to mimic simplified versions of the World Health Organization (WHO) syndromic case definitions ((4)). Specifically, ILI and ARI percentages were calculated as the number of reports of cough and/or fever in a given week divided by the total number reports received by participants for that same week. The weekly total RVDs count is defined as the sum of the number of positive tests for all respiratory viruses (influenza, SARS-CoV-2, RSV, and the aggregated sum of adenovirus, coronavirus, enterovirus/rhinovirus, hMPV, and parainfluenza) in a given week. The SARS-CoV-2 case numbers are available by week using the earliest date between onset date, specimen collection date, laboratory testing date, date reported to province or territory, or date reported to PHAC ((14)).

### Statistical analysis

Spearman correlation coefficients were calculated to compare self-reported ILI and self-reported ARI against the weekly total RVD count and the weekly total RVD count excluding SARS-CoV-2 detections. These were calculated over the entire study period and for the time periods of March 1, 2020, to March 6, 2021; March 7, 2021, to March 5, 2022; and March 6, 2022, to March 4, 2023. Pearson correlation coefficients were unsuitable for these datasets, due to the high proportion of outliers in total RVDs. Furthermore, Shapiro-Wilk tests indicated that the populations were not normally distributed.

## Results

### Participants

Between March 1, 2020, and March 4, 2023, FluWatchers consisted of 25,326 participants. The average response rate per week over the entire study period was 45% and ranged from 13% to 51%. However, the average weekly response rates per individual year (March 2020 to March 2021, March 2021 to March 2022, and March 2022 to March 2023) were 51%, 65%, and 65% respectively. Throughout the entire study period, a total of 10,716 participants (42%) had a response rate of 75% or greater; 3,121 participants (12%) had a response rate between 50%–74%; 5,907 participants (23%) had a response rate between 10%–49%; and 5,582 (22%) had a response rate of less than 10%. The majority of FluWatchers participants were female (63%), and less than 1% identified as gender diverse. FluWatchers participants were mainly located in Ontario (47%) and Québec (19%). The median year of birth of FluWatchers was 1977 (interquartile range, IQR=1963–1989). While the demographic characteristics of FluWatchers remained more or less similar throughout the study period, epidemiological trends varied year-by-year (**Table 1**). For instance, the proportion of FluWatchers reporting an absence from work or school increased from 24.4% in 2020–2021 to 47.5% in 2022–2023. The proportion of FluWatchers reporting healthcare-seeking behaviour varied throughout the study period, increasing from 25.2% to 40.7% between 2020–2021 and 2021–2022, before sharply decreasing to 18.3% in 2022–2023.

**Table 1:** FluWatchers participation profile

FluWatchers characteristics	2020–2023	2020–2021	2021–2022	2022–2023
Total number of participants	25,326	21,005	19,026	17,066
Average weekly response rate (%)	44.7 (12.5–51.9)	51.1 (15.1–61.5)	64.9 (58.4–69.1)	64.8 (58.2–70.9)
Average weekly absenteeism rate (%)	38.4 (2.1–62.1)	24.4 (2.1–42.4)	43.9 (31.5–55.7)	47.5 (37.8–62.1)
Average weekly healthcare utilization rate (%)	27.9 (2.3–53.8)	25.2 (2.3–41.5)	40.7 (22.3–53.4)	18.3 (9.2–53.8)
Proportion of participants responding to >75% of surveys (%)	42	56	63.5	58.5
Proportion participants reporting in <10% of surveys (%)	22	10	8.6	10.4
Average ILI (%)	0.8	0.3	0.4	1.7
Average ARI (%)	4.6	3.5	2.7	7.6
Median year of birth (IQR)	1977 IQR=1963–1989	1977 IQR=1963–1989	1974 IQR=1961–1987	1973 IQR=1960–1986
Proportion of female (%)	62.6	62.6	63.1	63.5
Proportion of male (%)	36.9	37.2	36.5	36
Proportion gender diverse (%)	0.5	0.2	0.4	0.5
Influenza vaccination rate (%)	61.3	68.5	62.1	69.8

Abbreviations: ARI, acute respiratory infection; ILI, influenza-like illness; IQR, interquartile range

### Seasonal respiratory virus pattern

**Figure 1:**
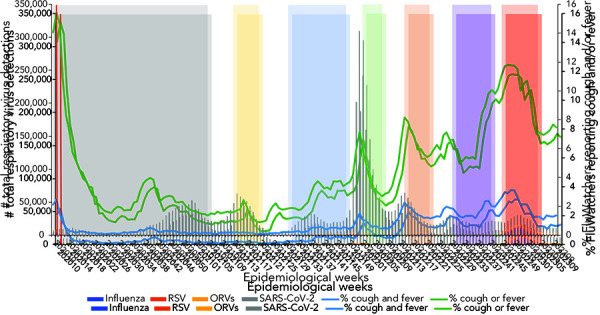
Total respiratory virus detections vs. influenza-like illness and acute respiratory infection reported by FluWatchers, March 1, 2020, to March 4, 2023, Canada^a,b,c,d,e,f,g,h^ Abbreviations: ORV, other respiratory viruses; RSV, respiratory syncytial virus; SARS-CoV-2; severe acute respiratory syndrome coronavirus 2 ^a^ The red dotted line represents the onset of public health measures related to COVID-19 ^b^ The grey shaded area (epidemiological weeks 2020-10 to 2021-04) represents the first wave of the COVID-19 pandemic ^c^ The yellow shaded area (epidemiological weeks 2021-14 to 2021-21) represents the Alpha, Beta, and Gamma variant predominance period of the COVID-19 pandemic ^d^ The blue shaded area (epidemiological weeks 2021-30 to 2021-48) represents the Delta variant predominance period of the COVID-19 pandemic ^e^ The green shaded area (epidemiological weeks 2022-01 to 2022-07) represents the Omicron BA.1 variant predominance period of the COVID-19 pandemic ^f^ The orange shaded area (epidemiological weeks 2022-14 to 2022-22) represents the Omicron BA.2 variant predominance period of the COVID-19 pandemic ^g^ The purple shaded area (epidemiological weeks 2022-29 to 2022-41) represents the Omicron BA.5 variant predominance period of the COVID-19 pandemic ^h^ The red shaded area (epidemiological weeks 2022-44 to 2023-03) represents the 2022–2023 influenza season

The temporal pattern in the proportion of FluWatchers respondents reporting ILI, ARI, and the weekly RVD count over the three years of the pandemic (March 2020 to March 2023) is shown in **Figure 1**.

The number of laboratory tests positive for seasonal respiratory viruses (influenza, adenovirus, coronavirus, enterovirus/rhinovirus, hMPV, parainfluenza, and RSV) demonstrated a rapidly declining end-of-season wave from March 8, 2020, to April 18, 2020, concurrent with the increase of the first epidemic wave of SARS-CoV-2.

**Figure 2:**
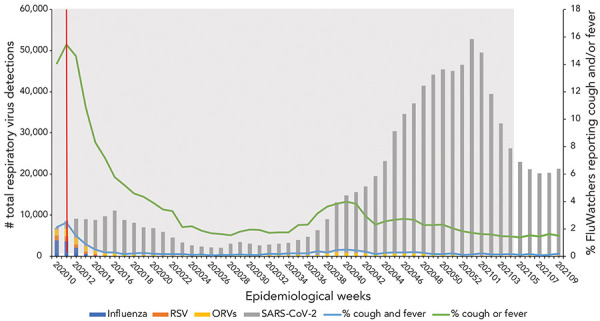
Total respiratory virus detections vs. influenza-like illness and acute respiratory infection reported by FluWatchers, March 1, 2020, to March 6, 2021, Canada^a,b^ Abbreviations: ORV, other respiratory viruses; RSV, respiratory syncytial virus; SARS-CoV-2; severe acute respiratory syndrome coronavirus 2 ^a^ The grey shaded area (epidemiological weeks 2020-10 to 2021-04) represents the first wave of the COVID-19 pandemic ^b^ The red dotted line represents the onset of public health measures related to COVID-19

**Figure 3:**
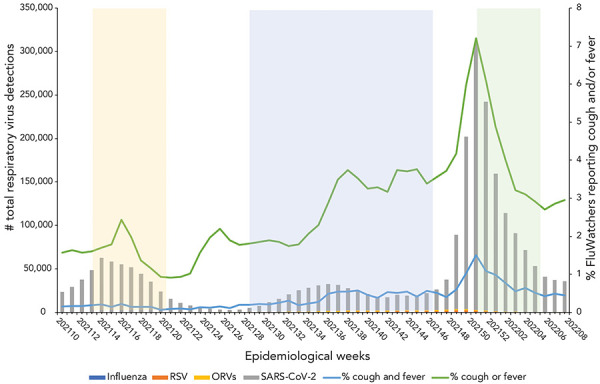
Total respiratory virus detections vs. influenza-like illness and acute respiratory infection reported by FluWatchers, March 7, 2021, to March 5, 2022, Canada^a,b,c^ Abbreviations: ORV, other respiratory viruses; RSV, respiratory syncytial virus; SARS-CoV-2; severe acute respiratory syndrome coronavirus 2 ^a^ The yellow shaded area (epidemiological weeks 2021-14 to 2021-21) represents the Alpha, Beta, and Gamma variant predominance period of the COVID-19 pandemic ^b^ The blue shaded area (epidemiological weeks 2021-30 to 2021-48) represents the Delta variant predominance period of the COVID-19 pandemic ^c^ The green shaded area (epidemiological weeks 2022-01 to 2022-07) represents the Omicron BA.1 variant predominance period of the COVID-19 pandemic

**Figure 4:**
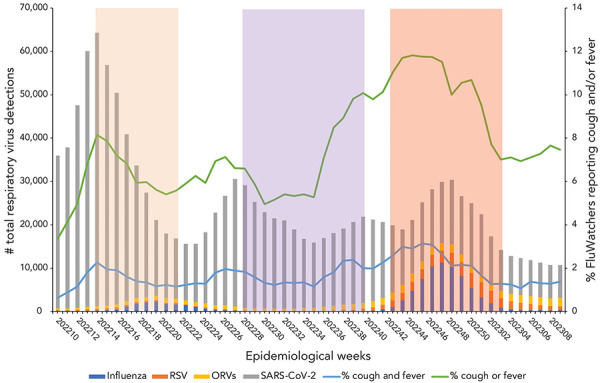
Total respiratory virus detections vs. influenza-like illness and acute respiratory infection reported by FluWatchers, March 6, 2022, to March 4, 2023, Canada^a,b,c^ Abbreviations: ORV, other respiratory viruses; RSV, respiratory syncytial virus; SARS-CoV-2; severe acute respiratory syndrome coronavirus 2 ^a^ The orange shaded area (epidemiological weeks 2022-14 to 2022-22) represents the Omicron BA.2 variant predominance period of the COVID-19 pandemic ^b^ The purple shaded area (epidemiological weeks 2022-29 to 2022-41) represents the Omicron BA.5 variant predominance period of the COVID-19 pandemic ^c^ The red shaded area (epidemiological weeks 2022-44 to 2023-03) represents the 2022–2023 influenza season

The SARS-CoV-2 case counts demonstrated that Canada experienced two epidemic waves over the first year of the COVID-19 pandemic (**Figure 2**). The first occurred in the spring of 2020, with case counts peaking mid-April, and the second in the autumn/winter of 2020–2021, with case counts peaking the last week of December. During the second year of the COVID-19 pandemic, Canada experienced three more epidemic waves (**Figure 3**). The first occurred in the spring of 2021, with case counts peaking around April 2021. The second wave began late in the summer of 2021, lasting until December 2022. This wave was quickly followed by the third epidemic wave of the second year of the pandemic, occurring in the early months of 2022 and marked by the predominant circulation of the Omicron BA.1 variant. During the third year of the COVID-19 pandemic, Canada experienced two final consecutive epidemic waves, mainly attributable to the circulation of Omicron variants (BA.2 and BA.5) (**Figure 4**). Both took place between March 2022 and October 2022, with the first peaking early in April, and the second peaking early in July. This year was also marked by the return of the circulation of ORVs. An early and intense resurgence of influenza A occurred in the fall of 2022, before subsiding shortly after January 2023 ((16)).

### Association between self-reported influenza-like illness and acute respiratory infection and laboratory-confirmed respiratory virus detections

The FluWatchers syndromic surveillance indicator—the percentage of participants meeting the ILI and the ARI case definitions—showed a similar pattern to that of respiratory virus detections. Both the ILI and ARI rates mirrored the sharp decline in percentage positive observed for seasonal respiratory viruses. No deceleration in the decline (or increase) corresponding to the rise in SARS-CoV-2 during the first epidemic wave (epidemiological weeks 2020-10 to 2020-24; March 2020 to June 2020) was observed.

The ILI rate remained flat during the typical inter-seasonal period during which influenza, other respiratory viruses, and SARS-CoV-2 curves were lower, with a mild signal/increase in the early fall (weeks September 6, 2020, to October 11, 2020) corresponding to an increase predominantly consisting of rhinovirus/enterovirus ((17)). The ILI rate fluctuated through the second and third waves of SARS-CoV-2 (November 2020 to June 2021) and the FluWatchers ARI rate captured trends in RVD case counts. The FluWatchers ARI tracked laboratory-confirmed RVDs accurately as of epidemiological week 2021-11. Self-reported ILI began to demonstrate accurate overlap with laboratory-confirmed RVDs as of September 2021. During the fourth wave of SARS-CoV-2 activity, self-reported ILI peaked around the same time as RVDs. The same applies to all subsequent RVDs spikes.

Despite these visual trends, there is insufficient statistical evidence of a significant correlation between self-reported ILI and the total RVDs including SARS-CoV-2 over the entire study period [ρ=0.16 (−0.0015, 0.30); *p*=0.05], as well as during the isolated period of March 2020 to March 2021 [ρ=0.18 (−0.11, 0.30); *p*=0.21] (**Table 2**). However, self-reported ILI showed a moderate correlation with total RVDs from March 2021 to March 2022 [ρ=0.51 (0.27, 0.69); *p*=1.02e-04] and a low correlation from March 2022 to March 2023 [ρ=0.34 (0.07, 0.57); *p*=0.01]. The same trends apply to self-reported ARI, where there was also no statistical evidence of a significant correlation between self-reported ARI and the total RVDs, including SARS-CoV-2, over the entire study period [ρ=−0.03 (−0.18, 0.13); *p*=0.72], and from March 2020 to March 2021 and from March 2022 to March 2023. However, from March 2021 to March 2022, self-reported ARI demonstrated a stronger correlation with total RVDs [ρ=0.40 (0.13, 0.61); *p*=3.53e-03] (Table 2).

**Table 2:** Spearman correlation results, influenza-like illness and acute respiratory infection reported by FluWatchers vs. total respiratory virus detections, including SARS-CoV-2

Year	ILI		ARI	
	ρ (95% CI)	*p*-value	ρ (95% CI)	*p*-value
2020–2023	0.16 (−0.0015, 0.30)	0.05	−0.03 (−0.18, 0.13)	0.72
2020–2021	0.18 (−0.11, 0.43)	0.21	−0.09 (−0.36, 0.19)	0.51
2021–2022	0.51 (0.27, 0.69)	1.02e-04	0.40 (0.13, 0.61)	3.53e-03
2022–2023	0.34 (0.07, 0.57)	0.01	0.01 (−0.27, 0.29)	0.92

Abbreviations: ARI, acute respiratory infection; CI, confidece interval; ILI, influenza-like illness

When SARS-CoV-2 detections are excluded from the total RVD counts, the correlation coefficients with self-reported ILI over the entire study period [ρ=0.87 (0.82, 0.90); *p*=3.12e-51] and for the first two isolated years of the pandemic are strong and statistically significant. Over the period of March 2022 to March 2023, the correlation between self-reported ILI and total RVDs excluding SARS-CoV-2 is moderate [ρ=0.37 (0.10, 0.59); *p*=7.47e-03]. However, self-reported ARI exhibits a strong and statistically significant correlation with total RVDs excluding SARS-CoV-2 over the entire study period [ρ=0.88 (0.83, 0.91); *p*=5.17e-54] and over each isolated year of the pandemic (**Table 3**).

**Table 3:** Spearman correlation results, influenza-like illness and acute respiratory infection reported by FluWatchers vs. total respiratory virus detections, excluding SARS-CoV-2

Year	ILI		ARI	
	ρ (95% CI)	*p*-value	ρ (95% CI)	*p*-value
2020–2023	0.87 (0.82, 0.90)	3.12e-51	0.88 (0.83, 0.91)	5.17e-54
2020–2021	0.84 (0.74, 0.91)	2.20e-15	0.85 (0.75, 0.91)	7.37e-16
2021–2022	0.83 (0.71, 0.90)	4.36e-14	0.88 (0.80, 0.93)	7.53e-18
2022–2023	0.37 (0.10, 0.59)	7.47e-03	0.70 (0.53, 0.82)	6.60e-09

Abbreviations: ARI, acute respiratory infection; CI, confidece interval; ILI, influenza-like illness

## Discussion

Overall, ILI and ARI rates followed a similar pattern as total RVDs throughout the study period, including throughout specific epidemic waves. The correlation between self-reported ILI and ARI and total RVDs, including SARS-CoV-2, was not statistically significant in the early pandemic but became moderate or low in subsequent years. However, when SARS-CoV-2 detections were excluded from the total respiratory virus counts, the correlation between self-reported ILI and total RVDs became strong and statistically significant for the entire study period and the early pandemic years. Self-reported ARI exhibited a strong and statistically significant correlation with total RVDs when SARS-CoV-2 was excluded, both for the entire study period and each isolated year of the pandemic.

Since its inception in 2015, the main objective of the FluWatchers program was to track the spread of influenza in Canada ((1)). However, over the years, program improvements have enabled the system to track the spread of other respiratory viruses, such as SARS-CoV-2, RSV, and others. This analysis provides valuable insight into how FluWatchers acts as a complement to traditional surveillance systems for non-specific respiratory virus surveillance. At the time of writing, this article is the first known publication comparing the performance of a participatory syndromic surveillance program's ILI data against laboratory detections of SARS-CoV-2, influenza, RSV, and other seasonal respiratory viruses over the three years of the COVID-19 pandemic.

The COVID-19 pandemic has highlighted the need for robust surveillance of respiratory viruses. The WHO mosaic framework for the surveillance of respiratory viruses of epidemic and pandemic potential has stated that it is “impossible to address the many complex needs of respiratory virus surveillance with a single surveillance system” ((11)), and as such, multiple systems need to function together in order to achieve specific surveillance objectives and targets. The framework specifies three main domains to classify surveillance approaches, and within each domain, both core and enhanced surveillance approaches are recommended. Most notably, syndromic surveillance is included as an enhanced surveillance approach in domains 1 and 2, and participatory surveillance is included as a surveillance innovation in all three domains ((11)).

• Domain 1: detection and assessment of an emerging or re-emerging respiratory virus

• Domain 2: monitor epidemiological characteristics of respiratory viruses in interpandemic periods

• Domain 3: informing use of human health intervention

Participatory surveillance is particularly touted for its ability to complement traditional surveillance by capturing information from individuals who may not seek care ((12,18)). Additionally, participatory surveillance may be able to identify peaks of respiratory virus activity earlier than sentinel or laboratory surveillance ((18)). This can be highlighted through visual assessment of Figure 1, wherein peaks in RVDs are typically preceded by peaks in self-reported ILI. For instance, during the 2022–2023 influenza season (Figure 4), total RVDs peaked in epidemiological week 2022-50 (week ending December 17, 2022), whereas self-reported ILI through FluWatchers peaked three to four weeks sooner (week 2022-48, ending December 3, 2022). Participatory surveillance is also useful in its ability to gather additional information on both healthcare seeking, testing, and vaccination behaviours in the population ((11)). FluWatchers collects valuable data, including whether an individual took time off work or school, or whether they consulted a healthcare professional in a given week ((13)). Additionally, FluWatchers is one of the only national programs that collects data on rapid antigen testing for COVID-19, including both numerator and denominator data. Despite the fact that participatory surveillance populations are typically not representative of the general population, which is the case in the FluWatchers population, their data provides great insight over periods of time ((9)). Participatory surveillance offers the advantage of effectively identifying and tracking community respiratory circulation.

This analysis is limited by various factors. A known weakness of participatory surveillance systems is the bias associated with participant self-selection into the program, as those who choose to participate are systematically different than those who do not ((19)). As such, participant populations in participatory syndromic surveillance systems tend to differ from the population they are intended to represent. The FluWatchers population differs from the Canadian population in terms of gender, age, and geographical distribution. This limits the extent to which age and geography-specific trends can be discussed. Paediatric population and elderly populations are underrepresented in FluWatchers data. These populations are often marked by particular community circulation trends, which may not be reflected in Canada's participatory syndromic surveillance. Additionally, the two populations being compared are fairly distinct. Those who seek testing for respiratory viruses, and therefore would be included in the total RVD dataset, are typically individuals who are already sick and seeking care. However, FluWatchers typically represent a subset of relatively healthy members of the general population ((12)). This can be demonstrated by the results obtained in the year one correlation analysis (March 2020 to March 2021). In the first year of the pandemic, total RVDs showed increases at several points, however, no associated increases in the percentage of FluWatchers reporting a cough and a fever were present. The SARS-CoV-2 cases in the first year of the pandemic were concentrated in outbreaks primarily occurring in long-term care facilities ((20)). The FluWatchers population is not adequately representative of this segment of the population ((13)), and thus FluWatchers data was not able to capture these spikes in SARS-CoV-2 activity. A large majority of Canadians were subject to public health measures during this phase in the pandemic ((21)). Due to the FluWatchers population's higher likelihood to engage in health promoting behaviours, there was a high probability that FluWatchers participants were adhering to these public health measures, thus limiting their exposure to SARS-CoV-2 and respiratory viruses in general. In the following year, namely throughout the Omicron wave, the community spread of SARS-CoV-2 increased sharply. The FluWatchers population appeared to have captured these spikes in activity (Figure 3 and Figure 4 and Spearman correlation results [ρ=0.51 [0.27, 0.69]; *p*=1.02e-04]).

Another limitation to address is the fact that total RVDs are sensitive to policy changes, most notably changes in testing practices. In the case of SARS-CoV-2 detections, asymptomatic testing for SARS-CoV-2 was a global common practice into the second year of the pandemic ((22)). FluWatchers is a syndromic surveillance system and would not capture asymptomatic cases, so this may have been a factor in the poor correlation during the first year of the pandemic. Additionally, a shift toward the use of rapid antigen tests after December 2021 in Canada and a reduction of widespread polymerase chain reaction (PCR) testing availability across many jurisdictions lead to a decline in the number of reported cases, which was likely not reflective of the actual prevalence of SARS-CoV-2 in the community ((14,23)). This artificial decline in SARS-CoV-2 detections may have contributed to the poor correlation with FluWatchers' self-reported ILI and ARI indicator, as FluWatchers data was still able to capture community circulation of all respiratory viruses, including SARS-CoV-2. This can be further demonstrated through the analysis of the correlation results excluding SARS-CoV-2. During the COVID-19 pandemic, testing eligibility for influenza and ORV did not change as frequently, nor as significantly, as those for SARS-CoV-2, as PCR testing for these viruses is most commonly performed among symptomatic individuals. This may explain why the Spearman correlations between total RVDs excluding SARS-CoV-2 and the self-reported ILI indicator were positive and statistically significant over the entire study period and in each individual year.

Influenza-like illness has been a long-standing syndromic case definition for influenza surveillance. While the WHO recommends ILI surveillance for COVID-19 syndromic surveillance ((24)), COVID-19 may present with a greater variety of symptoms other than cough or fever ((25)). Self-reported symptoms vary not only over the course of infection but also between individual cases and different age groups ((26)). Much remains to be understood about COVID-19, its symptomatology, and its extended impacts, notably encompassing long COVID and post-COVID symptoms. This may challenge the use of ILI or ARI case definitions for capturing COVID-19 community activity. The establishment of an optimal syndromic case definition will become a pertinent consideration once COVID-19 seasonality is established. This is especially true if ILI and ARI exhibit shortcomings in accurately capturing COVID-19 activity in the future.

## Conclusion

In summary, despite the correlation results obtained in the analysis, FluWatchers remains a valuable component of Canada's influenza monitoring strategy and will remain an important component in an integrative program for non-specific respiratory virus surveillance. FluWatchers self-reported ILI showed a moderate to strong correlation with total RVDs, and while this correlation decreased in strength and significance with the addition of SARS-CoV-2 data, a trend that is both expected and explained as discussed above, the association between the two data sources still existed. The concordance between respiratory detections and the proportion of FluWatchers reporting a cough and a fever is clearly demonstrated in the visual trends (Figure 1). FluWatchers data provides a point-in-time estimate of non-specific respiratory virus activity in the community, and can be used to detect periods of high or unusual circulation in near-real time within a respiratory season.
